# Long-Term Implantable Cardioverter Defibrillator Lead Dysfunction After Left Ventricular Assist Device Implantation

**DOI:** 10.1016/j.jacadv.2025.102258

**Published:** 2025-10-24

**Authors:** Tsukasa Oshima, Kohei Ishibashi, Kenichiro Yamagata, Nobuhiko Ueda, Toshihiro Nakamura, Satoshi Oka, Yuichiro Miyazaki, Akinori Wakamiya, Kenzaburo Nakajima, Yu Shimizu, Takuya Watanabe, Tsukasa Kamakura, Mitsuru Wada, Junichi Ishida, Yuko Inoue, Koji Miyamoto, Eisuke Amiya, Katsuhito Fujiu, Masaru Hatano, Yasumasa Tsukamoto, Takeshi Aiba, Satsuki Fukushima, Norihiko Takeda, Minoru Ono, Kengo Kusano

**Affiliations:** a Department of Cardiovascular Medicine, National Cerebral and Cardiovascular Center, Suita, Japan; b Department of Cardiovascular Medicine, The University of Tokyo Graduate School of Medicine, Bunkyo, Japan; c Department of Transplant Medicine, National Cerebral and Cardiovascular Center, Suita, Japan; d Department of Cardiac Surgery, National Cerebral and Cardiovascular Center, Suita, Japan; e Department of Cardiovascular Surgery, The University of Tokyo Graduate School of Medicine, Bunkyo, Japan

**Keywords:** implantable cardioverter defibrillator, lead dysfunction, left ventricular assist device

## Abstract

**Background:**

Implantable cardioverter defibrillator (ICD) lead dysfunction after left ventricular assist device (LVAD) implantation can occur, but only short-term outcomes have been reported.

**Objectives:**

We aimed to evaluate the long-term incidence, characteristics, and predictors of persistent ICD lead dysfunction after LVAD implantation.

**Methods:**

This was a retrospective multicenter study. All patients with a transvenous ICD lead at the time of LVAD implantation between January 1, 2011, and December 31, 2023, were enrolled. The primary endpoint was lead dysfunction. Risk factors for persistent compared to temporary lead dysfunction using a logistic analysis were determined.

**Results:**

One hundred and seventy patients (mean age: 48.0 ± 12.7 years) were analyzed. The median follow-up period was 46.2 (Q1-Q3: 32.3-61.4) months. Lead dysfunction was observed in 124 leads (72.9%), of which 60.4% (N = 75) occurred within a year after LVAD implantation. Of the 124 lead dysfunctions, 84 (67.7%) showed persistent dysfunction and 40 (32.3%) were temporary. Lead dysfunction occurring later than 2.1 months after LVAD implantation was a risk factor for persistent lead dysfunction (area under the curve: 0.673). Multivariable analysis identified lead dysfunction occurring later than 2.1 months after LVAD implantation as an independent risk factor for persistent lead dysfunction (OR: 4.67; 95% CI: 2.05-10.94; *P* < 0.001).

**Conclusions:**

In patients undergoing LVAD implantation, ICD lead dysfunction was observed in 72.9% after a mean follow-up of 46.2 months, of which two-thirds had persistence lead dysfunction. Later occurrence of lead dysfunction (>2 months after implant) may be a risk factor for persistent lead dysfunction.

Despite the development of treatments for heart failure (HF), including drug and cardiac electrical device therapies, some patients progress to end-stage disease and advanced HF.^1^, ^2^, ^3^, ^4^, ^5^ Left ventricular (LV) assist device (LVAD) implantation and heart transplantation may be options in patients with advanced HF who are refractory to optimal therapy.^4^^,^^6^

Many patients with LVADs have already been implanted with a pacemaker, implantable cardioverter defibrillator (ICD), or cardiac resynchronized therapy device before LVAD implantation; reportedly, approximately 80% of patients undergoing LVAD implantation already have an ICD.^7^ Given the physical proximity of LVAD and cardiac implantable electrical device (CIED) systems, there are issues related to the interactions between these devices.^8^, ^9^, ^10^

Previous studies have reported that the incidence of CIED lead dysfunction is only 2.3% to 3.0% over a median follow-up period of 4 years.^11^^,^^12^ The populations included in these studies were patients with CIEDs or ICDs; as such, they were not limited to patients with LVADs. In contrast, previous studies have shown a high incidence of CIED lead dysfunction in patients with LVADs, with a prevalence of 55% during a median follow-up period of 9.1 months.^13^ Some studies have reported that LVAD implantation is associated with a significant decrease in sensing amplitude and impedance and an increase in threshold.^14^, ^15^, ^16^ These results suggest a high incidence of lead dysfunction in patients with an LVAD. However, the follow-up periods in previous studies on patients with LVADs were quite short, with follow-up periods that were almost shorter than 1 year.^13^, ^14^, ^15^, ^16^

In Asia, due to the small number of heart donors and the increasing number of patients implanted with LVADs every year, the average waiting period before receiving heart transplantation after LVAD implantation is >900 days and growing.^17^^,^^18^ This waiting period is considerably longer than that in Western countries.^19^

Although LVAD typically served as a bridge-to-transplantation (BTT) or bridge-to-recovery option in the past, its use as destination therapy (DT) has increased.^20^ Compared to patients with LVAD for BTT or bridge to recovery, those with DT require LVAD support for much longer. Because the number of patients with LVAD on DT is estimated to increase annually, a higher incidence of lead dysfunction is expected.^21^ However, data on the long-term influence of LVAD on lead dysfunction are lacking, and will be needed in the future. In Japan, the period of LVAD usage is long, and obtaining long-term data can help evaluate this issue. Herein, we evaluated the incidence and risk factors for ICD lead dysfunction in patients with LVADs during a long-term follow-up period.

## Methods

### Study population

This was a retrospective, multicenter study (National Cerebral and Cardiovascular Center and the University of Tokyo Hospital). Consecutive patients who had a transvenous ICD lead and received a continuous-flow LVAD in these centers between January 1, 2011, and December 31, 2023, were assessed and followed until October 31, 2024. Patients aged <18 years were excluded, as in other studies.^13^^,^^14^^,^^16^ Patients without transvenous ICD leads before LVAD implantation were also excluded. LVADs included the DuraHeart (Terumo Heart, Inc), EVAHEART (Sun Medical Technology Research Corp), HVAD (Medtronic), HeartMate II and 3 (Abbott Laboratories), and Jarvik 2000 (Jarvik Heart Inc). The study protocol conformed to the Declaration of Helsinki and was reviewed and approved by the Institutional Ethics Committee, the National Cerebral and Cardiovascular Center (M26-150-19), and the Research Ethics Committee, Graduate School of Medicine and Faculty of Medicine, and the University of Tokyo (2650). This retrospective study analyzed anonymous data generated after patients agreed to undergo treatment; hence, an opt-out method was used to obtain informed consent.

### Data collection

We reviewed the medical records and interrogation reports of CIED recipients. Lead parameters, including right ventricular (RV) sensing, pacing thresholds, impedance, and high-voltage (HV) impedance, were collected at every outpatient clinic visit from the date of interrogation. Data were collected until the patients underwent CIEDs removal or until the ICD lead was discontinued.

### Outcomes: Persistent and temporary lead dysfunction

As previously reported,^13^^,^^16^ lead dysfunction was defined as the temporary or persistent presence of 1 or more of the following from baseline: decrease of R wave >50%, increase of threshold >50%, change of impedance >100 Ω, and change of HV impedance >10 Ω.

The temporary lead dysfunction was defined as lead dysfunction, followed by improvement as follows: sensing that decreased by >50% from baseline but subsequently increased to >50%, pacing threshold that increased by >50% but subsequently returned to <150%, impedance change >100 Ω with subsequent change within <100 Ω from the baseline value, and HV impedance change >10 Ω with subsequent change within <10 Ω from the baseline value.^16^ Persistent lead dysfunction was defined as a lead dysfunction that did not improve.

The time of occurrence and incidence of lead dysfunction were evaluated. Lead dysfunction was divided into 2 groups (temporary and persistent dysfunction, defined previously), and their characteristics were evaluated. Multivariable logistic analysis was used to evaluate the predictive risk factors of persistent compared to temporary lead dysfunction. The parameter changes in the leads were also analyzed in patients who could be followed for more than 2 years. Multivariable Cox regression was used to examine for determinants of persistent lead dysfunction among all patients included in this study.

### Outcomes: Clinical event

The adverse clinical outcome or interest was mortality from any cause. Clinical outcomes were monitored throughout hospital admissions and every 3 to 6 months. Most of the patients included in this study were followed through in-person visits to our outpatient clinic. However, the rest were followed at other institutions, and follow-up information was obtained through referral letters or medical record exchanges.

### Statistical analysis

Univariable analyses were performed for continuous and categorical variables. Continuous variables with a normal distribution are presented as the mean ± SD or the median (25th-75th percentiles [Q1-Q3]) if skewed distribution. The normality of continuous variables was assessed using the Shapiro-Wilk test. Categorical variables are presented as frequencies (percentages). Continuous data groups were compared using Student's *t*-test or Wilcoxon rank-sum test for normally distributed and skewed data, respectively. Categorical data groups were compared using the chi-square or Fisher exact test as appropriate. Multivariable logistic analysis was performed to compare patients with persistent vs temporary lead dysfunction using variables with a *P* value <0.05 in univariable analysis. The results are expressed as ORs with 95% CIs. The linearity assumption was evaluated by plotting predicted logit values against the continuous predictors. Receiver operating characteristic (ROC) curve analysis was performed to define cutoff values for the time between lead dysfunction and LVAD implantation using the Youden index method. The area under the curve (AUC) was used to quantify diagnostic accuracy. The AUC was compared to 0.5 using the DeLong test. To further assess the relationship between time to lead dysfunction and the probability of persistent dysfunction, a restricted cubic spline analysis with 3 knots was performed using logistic regression. This approach allowed flexible modeling of potential nonlinear associations. Model fit was evaluated by likelihood ratio tests. The Kruskal-Wallis test was used to compare the medians among 3 groups. When it indicated significant differences, post hoc pairwise comparisons were conducted using the Dunn multiple comparison test with Bonferroni correction to identify which groups differed. A Cox proportional hazards regression model was used to analyze lead dysfunction rates across all patients over time. Patients who died before experiencing the event of interest were censored at the time of death. Variables with *P* values <0.10 in univariable analysis and clinically relevant variables were entered into the multivariable Cox model to identify the independent predictors of persistent lead dysfunction compared to no persistent lead dysfunction in all patients. Results are expressed as HRs with 95% CIs. The proportional hazards assumption was assessed using Schoenfeld residuals. A *P* value >0.05 was considered to indicate no violation of the proportional hazards assumption. All-cause mortality stratified by lead dysfunction was evaluated using the Kaplan-Meier method and compared using the log-rank test. The results were considered statistically significant at *P* < 0.05. All statistical analyses were performed using JMP software (version 18.2.1; SAS Institute Inc), except for the ROC analysis, Gray test, evaluation of the linearity assumption, and assessment of the proportional hazards assumption, which were performed using R software (version 4.5.1; The R Foundation for Statistical Computing).

## Results

### Baseline characteristics

**Figure 1:**
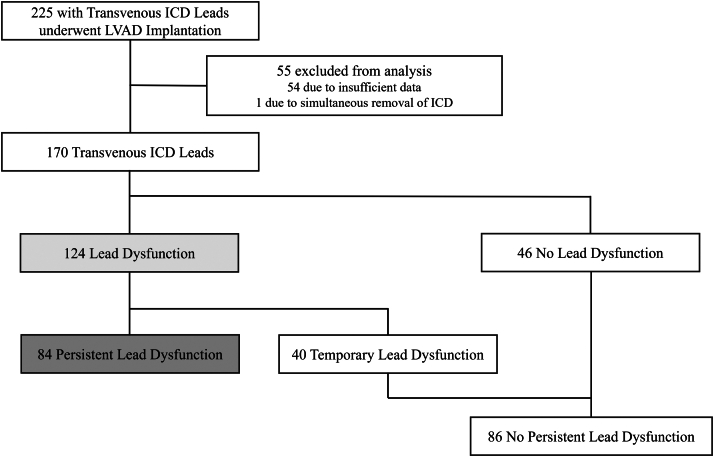
**Recruitment Strategy of Our Study** ICD = implantable cardioverter defibrillator; LVAD = left ventricular assist device.

Two hundred and twenty-five patients with a transvenous ICD lead underwent LVAD implantation between January 1, 2011, and December 31, 2023. Of these, 54 were excluded due to insufficient data. One patient had the ICD removed at the same time as LVAD implantation and was excluded (Figure 1).

A total of 170 patients were analyzed and the median follow-up duration after LVAD implantation was 46.2 (Q1-Q3: 32.3-61.4) months. The baseline characteristics are shown in Table 1.

**Table 1:** Baseline Demographics (N = 170)

Age, y	48.0 ± 12.7
Female	51 (30.0)
Body mass index, kg/m^2^	20.9 (18.7-23.1)
Left ventricular ejection fraction, %	17.8 (12.9-23.0)
Left ventricular end-diastolic diameter, mm	71.5 ± 14.4
Interventricular septal thickness, mm	7 (6-8)
Hypertension	7 (4.1)
Diabetes mellitus	28 (16.5)
Chronic kidney disease	89 (52.4)
Estimated glomerular filtration rate, mL/min/1.73 m^2^	58.8 ± 23.8
Nonischemic cardiomyopathy	159 (93.5)
Prior sternotomy	30 (17.6)
Secondary prevention	47 (29.6)
Cardiac resynchronization therapy	129 (75.9)
Medtronic implantable cardioverter defibrillator lead	86 (50.6)
Abbott implantable cardioverter defibrillator lead	38 (22.4)
Boston implantable cardioverter defibrillator lead	28 (16.4)
Biotronic implantable cardioverter defibrillator lead	17 (10.0)
Sorin implantable cardioverter defibrillator lead	1 (0.06)
Intermacs score	
1	21 (12.4)
2	34 (20.0)
3	98 (57.6)
4	17 (10.0)
Bridge to transplantation	153 (90.0)
Time from lead implantation to left ventricular assist device, months	30.5 (11.6-57.2)
Pacing threshold, V/0.4 ms	0.875 (0.625-1.110)
Sense, mV	9.93 ± 6.03
Right ventricular impedance, Ω	418 (361-477)
High voltage impedance, Ω	52 (42-63)
Type of left ventricular assist device	
HeartMate 3	46 (27.1)
HeartMate Ⅱ	72 (42.3)
Jarvik2000	14 (8.2)
HVAD	16 (9.4)
EVAHEART	18 (10.6)
DuraHeart	4 (2.4)
Concomitant cardiac surgery	
Tricuspid valve	57 (33.5)
Mitral valve	20 (11.8)
Aortic valve	22 (12.9)
Unplanned return to the operating room	29 (17.0)

Values are median (Q1-Q3), mean ± SD, or n (%).

The mean age was 48.0 ± 12.7 years, 30.0% (N = 51) patients were female, and 93.5% (N = 159) had nonischemic cardiomyopathy. One hundred and fifty-three patients (90.0%) were implanted with an LVAD as BTT. The median time between the previous ICD lead implantation and LVAD implantation was 30.5 (Q1-Q3: 11.6-57.2) months. Before LVAD implantation, the mean RV sensing amplitude was 9.93 ± 6.03 mV, the median pacing threshold was 0.875 (Q1-Q3: 0.625-1.110) V/0.4 ms, median impedance was 418 (Q1-Q3: 361-477) Ω, and median HV impedance was 52 (Q1-Q3: 42-63) Ω.

### Lead dysfunction

**Figure 2:**
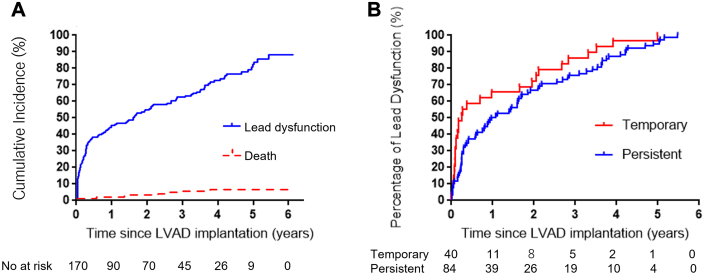
**Timing of Lead Dysfunction** (A) Shows the frequency of lead dysfunction among all 170 included leads. (B) Shows the timing of lead dysfunction in persistent (N = 84) and temporary (N = 40) leads. Abbreviations as in Figure 1.

During follow-up, a total of 124 patients (72.9%) experienced at least 1 criterion of significant lead dysfunction (Figure 2A): decrease of sensing >50% occurred in 66 leads (38.8%), worsening of pacing threshold >50% occurred in 21 leads (12.3%), the change of impedance >100 Ω occurred in 62 leads (36.4%), and change of HV impedance >10 Ω occurred in 76 leads (44.7%). Among the 124 leads with lead dysfunction, 60.4% (N = 75) occurred within a year of implantation, and 12.1% (N = 15), 8.9% (N = 11), and 18.6% (N = 23) occurred between 1 and 2, 2 and 3, and >3 years after LVAD implantation, respectively.

**Figure 3:**
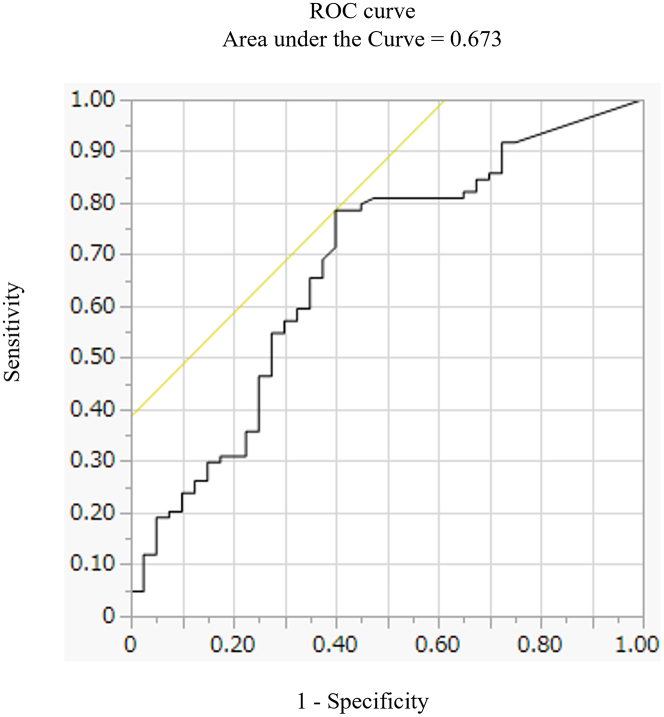
**Area Under the Receiver Operating Characteristic Curve for Lead Dysfunction** ROC analysis showed lead dysfunction occurring more than 2.1 months after LVAD implantation was predictive of persistent (N = 84) compared to temporary (N = 40) lead dysfunction (AUC, 0.673; *P* = 0.044; sensitivity, 0.785; specificity, 0.600). The 95% confidence intervals for AUC, sensitivity, and specificity were 0.57 to 0.78, 0.71 to 0.86, and 0.60 to 0.90, respectively. ROC=receiver operating characteristics; other abbreviations as in Figure 1.

Among the 124 leads with lead dysfunction, 67.7% (N = 84) and 32.3% (N = 40) were in the persistent and temporary dysfunction groups, respectively (Figure 1). Of the 170 leads included, 49.6% (N = 84) showed persistent lead dysfunction. The differences in the backgrounds of patients with persistent and temporary lead dysfunction are shown in Table 2. There were no significant differences in echocardiographic parameters, such as LV ejection fraction, comorbidities, type of LVAD, and ICD lead manufacturer. In contrast, the frequency of BTT was significantly higher in persistent dysfunction group (92.9% vs 80.0%; *P* = 0.041). The frequency of HeartMate II was also significantly higher in persistent dysfunction group (48.8% vs 30.0%; *P* = 0.045). The median occurrence time of lead dysfunction after LVAD implantation was significantly later in persistent lead dysfunction group than temporary lead dysfunction group (10.7 [Q1-Q3: 2.68-33.9] months vs 1.43 [Q1-Q3: 0.033-17.9] months; *P* = 0.001). ROC analysis demonstrated that the occurrence of lead dysfunction more than 2.1 months after LVAD implantation was predicted lead dysfunction, with an AUC of 0.673 (95% CI: 0.57-0.78; *P* = 0.044), sensitivity of 78.5% (95% CI: 0.71-0.86), and specificity of 60.0% (95% CI: 0.60-0.90) (Figure 3). The frequency of lead dysfunction after 2.1 months was significantly higher in the persistent dysfunction group than in the temporary dysfunction group (77.3% vs 40.0%; *P* < 0.001). After multivariable analysis, only an occurrence time later than 2.1 months after LVAD implantation was an independent risk factor for persistent lead dysfunction (OR: 4.67; 95% CI: 2.05-10.94; *P* < 0.001) (Table 3). To further examine the association between timing and risk, we performed a restricted cubic spline analysis, which showed a statistically significant nonlinear relationship between time to lead dysfunction and the probability of persistent dysfunction (likelihood ratio test, *P* = 0.025).

**Table 2:** Difference in Baseline Characteristics Between Patients With No Lead Temporary and Persistent Lead Dysfunction

	All Lead Dysfunctions (n = 124)	Persistent Dysfunction Group (n = 84)	Temporary Dysfunction Group (n = 40)	*P* Value
Age, y	48.5 ± 12.5	47.6 ± 13.1	50.2 ± 11.0	0.26
Female	38 (30.7)	27 (32.1)	11 (27.5)	0.60
Body mass index, kg/m^2^	21.0 ± 3.57	20.9 ± 3.26	21.0 ± 4.18	0.96
LVEF, %	17.8 ± 7.5	18.1 ± 8.1	17.1 ± 6.2	0.75
LVEDD, mm	72.3 ± 15.1	72.7 ± 15.6	71.5 ± 14.3	0.69
IVST, mm	6.76 ± 1.71	6.59 ± 1.75	7.10 ± 1.58	0.11
Hypertension	5 (4.0)	3 (3.6)	2 (5.0)	0.66
Diabetes mellitus	20 (16.1)	13 (15.5)	7 (17.5)	0.78
Chronic kidney disease	67 (54.0)	44 (52.4)	23 (57.5)	0.70
eGFR, mL/min/1.73 m^2^	58.4 ± 23.1	59.4 ± 23.7	56.0 ± 21.8	0.42
Nonischemic cardiomyopathy	115 (92.7)	77 (91.7)	38 (95.0)	0.72
Prior sternotomy	23 (18.6)	18 (21.4)	5 (12.5)	0.32
Secondary prevention	36 (29.0)	27 (32.1)	9 (22.5)	0.26
Cardiac resynchronization therapy	93 (75.0)	62 (73.8)	31 (77.5)	0.66
ICD lead manufacturer				
Medtronic ICD lead	63 (50.8)	44 (52.4)	19 (47.5)	0.61
Abbott ICD lead	27 (21.7)	17 (20.2)	10 (25.0)	0.55
Boston ICD lead	21 (16.9)	14 (16.7)	7 (17.5)	0.91
Biotronic ICD lead	12 (9.6)	8 (9.5)	4 (10.0)	1.00
Sorin ICD lead	1 (0.8)	1 (1.2)	0 (0.0)	1.00
Intermacs score				0.95
1	16 (12.9)	10 (11.9)	6 (15.0)	
2	23 (18.6)	15 (17.9)	8 (20.0)	
3	72 (58.1)	50 (59.5)	22 (55.0)	
4	13 (10.5)	6 (10.7)	4 (10.0)	
Bridge to transplantation	110 (88.7)	78 (92.9)	32 (80.0)	0.041
Time from lead implantation to LVAD, months	32.0 (15.9-63.1)	31.1 (14.0-57.4)	39.9 (18.2-68.1)	0.27
Type of LVAD				0.27
HeartMate 3	38 (30.6)	25 (29.8)	13 (32.5)	
HeartMate Ⅱ	53 (42.7)	41 (48.8)	12 (30.0)	
Jarvik2000	9 (7.3)	4 (4.8)	5 (12.5)	
HVAD	10 (8.1)	6 (7.1)	4 (10.0)	
EVAHEART	12 (9.7)	7 (8.3)	5 (12.5)	
DuraHeart	2 (1.6)	1 (1.2)	1 (2.5)	
Concomitant cardiac surgery	52 (41.9)	34 (40.5)	18 (45.0)	0.63
Unplanned return to the operating room	14 (11.3)	11 (13.1)	3 (7.5)	0.55
Time from LVAD to lead dysfunction, months	5.03 (0.941-26.0)	10.7 (2.68-33.9)	1.43 (0.033-17.9)	0.002
Time from LVAD to lead dysfunction >2.1 months	81 (65.3)	65 (77.4)	16 (40.0)	<0.001

Values are median (Q1-Q3), mean ± SD, or n (%). The *P* values were calculated by comparing the persistent and temporary dysfunction groups.

eGFR = estimated glomerular filtration rate; ICD = implantable cardioverter defibrillator; IVST = interventricular septal thickness; LVAD = left ventricular assist device; LVEDD = left ventricular end-diastolic diameter; LVEF = left ventricular ejection fraction.

**Table 3:** Univariable and Multivariable Models for Predictive Risk Factors of Persistent (N = 84) Compared to Temporary (N = 40) Lead Dysfunction

	Univariable Analysis		Multivariable Analysis	
	OR (95% CI)	*P* Value	OR (95% CI)	*P* Value
Age per 1-y increase	0.983 (0.951-1.014)	0.28		
Female	1.249 (0.552-2.949)	0.60		
Body mass index per 1-kg/m^2^ increase	0.996 (0.897-1.110)	0.95		
LVEF per 1% increase	1.020 (0.970-1.079)	0.45		
LVEDD per 1-mm increase	1.005 (0.980-1.031)	0.70		
IVST per 1-mm increase	0.840 (0.663-1.048)	0.13		
Hypertension	0.703 (0.112-5.508)	0.66		
Diabetes mellitus	0.863 (0.322-2.481)	0.78		
Chronic kidney disease	0.813 (0.377-1.732)	0.70		
eGFR per 1 mL/min/1.73 m^2^ increase	1.007 (0.990-1.025)	0.42		
Non ischemic cardiomyopathy	0.578 (0.084-2.533)	0.72		
Prior sternotomy	1.909 (0.693-6.120)	0.22		
Secondary prevention	1.631 (0.700-4.069)	0.26		
Cardiac resynchronization therapy	0.818 (0.324-1.943)	0.66		
ICD lead manufacturer				
Medtronic ICD lead	1.216 (0.572-2.584)	0.61		
Abbott ICD lead	0.761 (0.312-1.857)	0.55		
Boston ICD lead	0.943 (0.348-2.556)	0.91		
Biotronic ICD lead	0.947 (0.268-3.353)	1.00		
Intermacs score				
1	0.766 (0.257-2.279)	0.63		
2	0.870 (0.335-2.260)	0.78		
3	1.203 (0.563-2.573)	0.63		
4	1.080 (0.312-3.743)	1.00		
Bridge to transplantation	**3.250 (1.049-10.600)**	**0.041**	2.461 (0.698-9.025)	0.16
Time from lead implantation to LVAD	0.993 (0.984-1.001)	0.12		
Type of LVAD				
HeartMate 3	0.880 (0.394-2.013)	0.76		
HeartMate Ⅱ	**2.225 (1.016-5.087)**	**0.045**	1.539 (0.639-3.786)	0.34
Jarvik2000	0.350 (0.082-1.400)	0.15		
HVAD	0.692 (0.186-2.847)	0.73		
EVAHEART	0.636 (0.189-2.280)	0.47		
DuaHeart	0.470 (0.029-7.710)	0.54		
Concomitant cardiac surgery	0.831 (0.389-1.778)	0.63		
Unplanned return to the operating room	1.858 (0.541-8.583)	0.55		
Time from LVAD to lead dysfunction >2.1 months	**5.131 (2.308-11.817)**	**<0.001**	**4.666 (2.054-10.940)**	**<0.001**

Abbreviations as in Table 2.

### Changes in lead parameters over time

**Figure 4:**
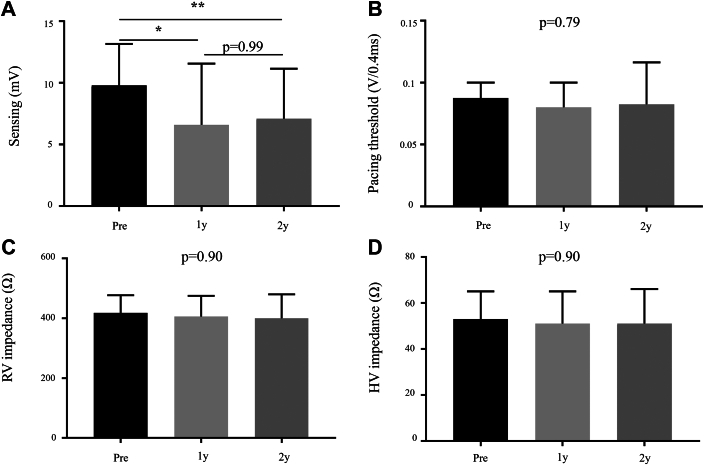
**Changes of Each Lead Parameter** (A) Sensing. (B) Pacing threshold. (C) Right ventricular impedance. (D) High-voltage impedance. Comparison of the individual lead parameters before (pre) LVAD implantation, one (1y) and 2 years (2y) after LVAD implantation during the follow-up period. The Kruskal-Wallis test was used to compare the medians among 3 groups. When the Kruskal-Wallis test indicated significant differences, post hoc pairwise comparisons were conducted using Dunn’s multiple comparison test with Bonferroni correction to identify which groups differed. When the Kruskal-Wallis test indicated no significant differences, only *P* values from the Kruskal-Wallis test were reported. Statistical significance is indicated as follows: ∗*P* < 0.05, ∗∗*P* < 0.01. HV = high-voltage; RV = right ventricular.

The changes in each parameter before and 1 and 2 years after LVAD implantation are shown in Figure 4. Among the 170 leads, 137 could be followed for >2 years. The median RV sensing amplitude decreased significantly 1 year after LVAD implantation (9.8 [Q1-Q3: 5.5-13.0] vs 6.6 [Q1-Q3: 4.3-11.5] mV; *P* = 0.012) (Figure 4A). However, there was no significant difference between 1 and 2 years after LVAD implantation (6.6 [Q1-Q3: 4.3-11.5] vs 7.1 [Q1-Q3: 4.1-11.1] mV; *P* = 0.99). In contrast, the pacing threshold (*P* = 0.79), impedance (*P* = 0.60), and HV impedance (*P* = 0.90) did not show significant differences (Figures 4B to 4D): The median pacing threshold changed from 0.875 (Q1-Q3: 0.625-1.000) to 0.800 (Q1-Q3: 0.625-1.000) and 0.825 (Q1-Q3: 0.700-1.125) V/0.4 ms, impedance changed from 418 (Q1-Q3: 380-476) to 406 (Q1-Q3: 361-475) and 400 (Q1-Q3: 361-480) Ω, and HV impedance changed from 53 (Q1-Q3: 42-65) to 51 (Q1-Q3: 42-65) and 51 (Q1-Q3: 43-66) Ω, 1 and 2 years after LVAD implantation, respectively.

### Predictors of persistent lead dysfunction in all icd leads

Using all 170 patients, multivariable Cox regression analysis showed that female sex (HR: 1.64; 95% CI: 1.03-2.63; *P* = 0.039), ischemic cardiomyopathy (ICM) (HR: 2.30; 95% CI: 1.04-5.07; *P* = 0.039), and HeartMate 3 (HR: 1.64; 95% CI: 1.01-2.64; *P* = 0.044) were independent risk factors for persistent lead dysfunction (Supplemental Table 1).

### Clinical outcomes related to lead dysfunction

**Figure 5:**
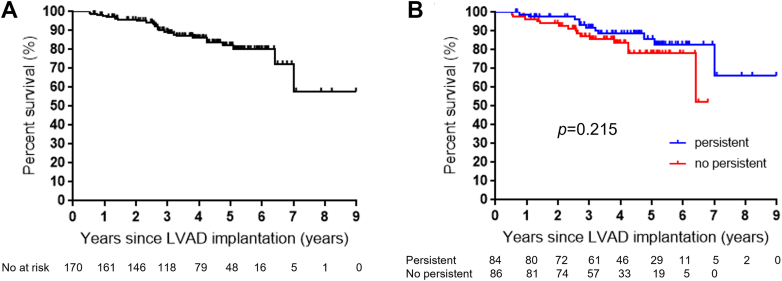
**Survival After Left Ventricular Assist Device Implantation** (A) Kaplan-Meier curve of all included patients (N = 170). (B) Comparison of time to death after LVAD implantation between patients in persistent and no persistent lead dysfunction group (N = 84 vs 86). There was no significant difference between these groups according to the log-rank test (*P* = 0.22). Abbreviations as in Figure 1.

Among 170 patients, 26 died during a median of 46.2 (Q1-Q3: 32.3-61.4) months of follow-up, including 10 patients due to HF, 9 patients due to cerebral hemorrhage, 2 patients due to cerebral infarction, 3 patients due to gastrointestinal hemorrhage, and 2 patients due to infection (Figure 5A). Of 84 patients in the persistent lead dysfunction group, 11 (13.1%) died. In contrast, of the 86 patients in the nonpersistent lead dysfunction group, 15 (17.4%) died. There was no significant difference in survival between groups (*P* = 0.22) (Figure 5B).

## Discussion

We evaluated ICD lead dysfunction in patients after LVAD implantation. The number of patients included in our study was larger, and the duration of follow-up was much longer than those in previous studies evaluating ICD lead dysfunction after LVAD implantation. Our main findings are as follows. First, lead dysfunction occurred in 124 leads (72.9%), of which 32.3% (40 leads) were temporary. Of the 170 leads, 49.6% (N = 84) exhibited persistent dysfunction. Second, approximately 60% of lead dysfunction was observed within 1 year of LVAD implantation; however, it was also observed continuously for several years. Third, later occurrence of lead dysfunction may be related to persistent lead dysfunction (Central Illustration). Fourth, among the 170 leads included, female sex, ICM, and HeartMate 3 implantation were independent predictive risk factors for persistent lead dysfunction.

**Central Illustration:**
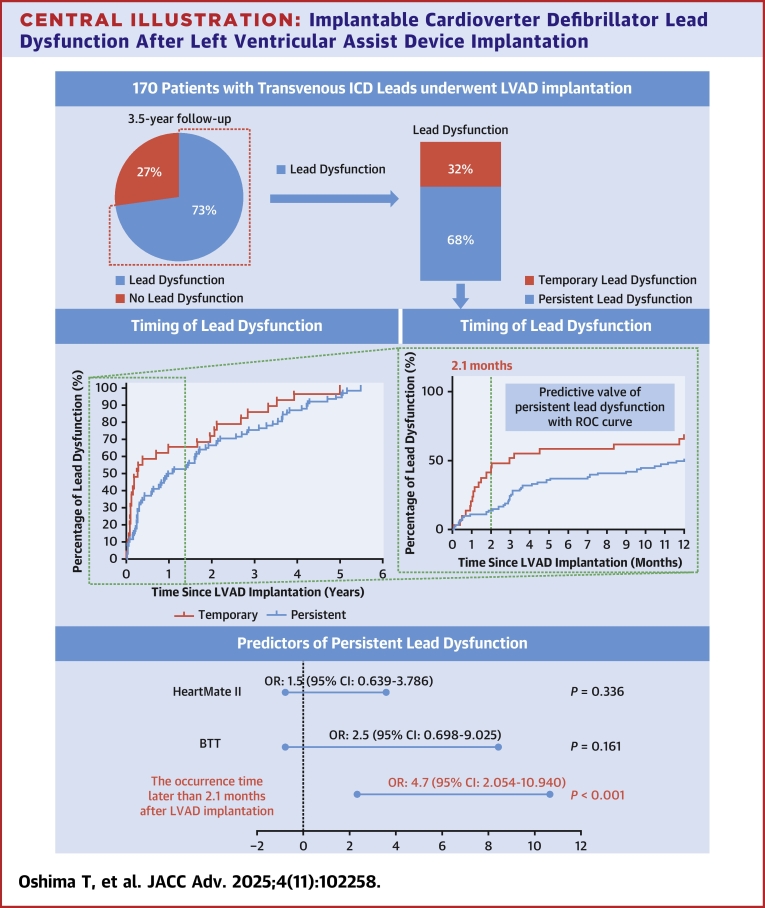
**Implantable Cardioverter Defibrillator Lead Dysfunction After Left Ventricular Assist Device Implantation** BTT = bridge to transplantation; ICD = implantable cardioverter defibrillator; LVAD = left ventricular assist device; ROC = receiver operating characteristics.

As the number of heart donors is limited and the number of patients implanted LVAD as BTT has been increasing, the duration of LVAD support until they can receive a heart transplantation should become longer.^22^ In addition, patients who have an implanted LVAD as a DT would require lifelong LVAD support. As LVAD support durations will increase, it is necessary to better understand and manage long-term LVAD support. Approximately 80% of the patients who underwent LVAD implantation had already been implanted with an ICD, and previous studies have reported several types of problems between LVAD and ICD, such as noise resulting in inappropriate shock and telemetry interference.^7^^,^^23^ We need to manage the problems caused by interactions between ICDs and LVADs, and our long-term research following up for longer durations than previous studies will have a significant impact.^9^

Few studies have evaluated ICD lead dysfunction after LVAD implantation.^13^, ^14^, ^15^, ^16^^,^^24^^,^^25^ These follow-up periods were almost within 1 year, and the maximum number of patients included in these studies was 122, as reported by Galand et al.^13^ Therefore, the number of patients included in our study was the largest to date, and the follow-up period was much longer than those in other studies, which allowed us to perform a more detailed analysis.^13^ In addition, including a larger number of patients, allowed us to divide lead dysfunction into 2 groups: persistent and temporary. Some lead dysfunctions are temporary, and persistent lead dysfunction sometimes requires invasive treatments such as lead extraction, some of which can be fatal.^26^ Therefore, it is important to focus on persistent lead dysfunction.

Galand et al. reported that the incidence of CIED lead dysfunction in patients with LVADs was 55% during a median follow-up period of 9.1 months.^13^ The incidence of lead dysfunction in our study was 72.9% during the median follow-up period of 46.2 months. We were able to show that lead dysfunction was seen even after 1 year of LVAD implantation, suggesting that we had better pay continuous attention to lead conditions. Moreover, with a longer follow-up duration, we found that one-third of lead dysfunctions were reversible.

Inflammation, edema, micro dislodgement, removal of the apical tissue core, and a left-sided shift of the ventricular septum are the causes of lead parameter changes, as discussed in previous studies.^13^^,^^16^ However, its exact mechanism remains unknown. We found that late occurrence of lead dysfunction may be a factor in persistent lead dysfunction. Soon after LVAD implantation, the inflammatory response persists and is relieved gradually. For example, a previous study showed that proinflammatory mediators such as interleukin-6, -8, and complement C3a were still elevated above normal levels for up to 6 weeks after LVAD implantation, although they gradually decreased to normal levels.^27^ Yu et al. reported that it took 5 months for C-reactive protein elevation to normalize after LVAD implantation, albeit this finding was in pediatric patients.^28^ Postoperative myocardial inflammation and edema can interfere with the electrode–myocardial interface, increase pacing thresholds, and reduce sensing amplitude.^29^ Moreover, the mechanical effects of LV unloading may induce a leftward septal shift, altering the lead position and contact pressure.^30^ In addition, apical coring for LVAD cannulation may remove myocardial tissue adjacent to the lead tip, leading to reduced sensing amplitude and increased capture threshold. Therefore, early changes in lead parameters may reflect transient physiological responses, whereas late or persistent dysfunctions may suggest irreversible structural alterations.

We found that female sex, ICM, and HeartMate 3 were independent predictive risk factors for persistent lead dysfunction among all included leads. This is the first study to identify the factors associated with persistent lead dysfunction in patients with LVAD.

Female sex has been reported as a risk factor for right HF and the need for RV assist devices after LVAD implantation.^31^^,^^32^ However, lead dysfunction in female patients has not been discussed in previous studies. As a sex difference of cardiovascular disease, higher inflammation or autoimmune disease in female has been reported.^31^ Immune responses are higher in females than in males, which has also been confirmed by evaluating inflammatory gene expression in human hearts.^33^^,^^34^ Thus, the estimated mechanism underlying the greater risk of persistent lead dysfunction in women is that they exhibit stronger inflammatory responses to LVAD implantation, resulting in persistently higher inflammation in the heart and lead parameter changes, such as decreased sensing. A smaller body size would be another cause, which limits LVAD implantation and potentially increases device–device interactions. Sex-related anatomical and electrophysiological differences, such as smaller heart size, thinner myocardium, and differences in myocardial fibrosis patterns, may influence lead–tissue interactions and affect pacing and sensing stability.^35^ These anatomical and electrophysiological differences may contribute to increased pacing thresholds or decreased sensing amplitudes after LVAD implantation.

We also found that ICM was an independent risk factor for persistent lead dysfunction. Previous studies have reported that patients with ICM may benefit less from cardiac resynchronized therapy than those with nonischemic cardiomyopathy, as indicated by a smaller improvement in the LV ejection fraction and higher mortality in the ICM group.^36^, ^37^, ^38^ The myocardial substrate in patients with ICM is thought to be less amenable to resynchronization.^38^ Rizzello et al. assessed the relationship between myocardial viability and future reverse remodeling using dobutamine stress testing in patients with ICM, finding that a substantial amount of viable myocardium prevented ongoing LV remodeling after revascularization.^39^ Moreover, ICM is characterized by transmural scar formation, heterogeneous conduction, and reduced myocardial viability, all of which can impair the stability and performance of the implanted leads. Extensive myocardial fibrosis may limit the ability of leads to maintain effective contact with excitable tissues, leading to persistent dysfunction. These studies suggest that ICM is a progressive and ongoing disease, which is compatible with our finding that ICM is a risk factor for persistent lead dysfunction.

HeartMate 3 is a magnetically levitated centrifugal continuous-flow pump engineered to avert pump thrombosis, which was often seen as a problem with the HeartMate II and HVAD.^40^ Pump thrombosis requires a surgical pump exchange, resulting in substantial complications and increased cost.^41^

No study has assessed the relationship between HeartMate 3 and ICD lead dysfunction. Although the exact mechanism remains unclear, we propose the following possible explanations based on clinical observations and the unique characteristics of HeartMate 3. Resection of the LV apex for implantation is a feature of HeartMate 3 implantation.^42^ Resection can decrease the amount of LV myocardium and increase the risk of tissue injury and microdislodgement of the shock lead. However, as HeartMate II, similar to HeartMate 3, requires resection, another mechanism may be involved in inducing lead dysfunction. Although HeartMate II is implanted in the peritoneum, HeartMate 3 can be implanted in the intrapericardial space.^40^ Therefore, the device itself is closer to heart and heart deformation caused by upward compression would be more likely to be induced. Moreover, because HeartMate 3 is implanted in the intrapericardial space, a foreign body reaction or inflammation may persist in the pericardial space, resulting in an elevated threshold or decrease in sensing. Although HVAD can also be implanted in the intrapericardial space, heart deformation caused by upward compression is more likely to be induced by HeartMate 3 because HeartMate 3 is thicker than HVAD. Combined with such features, HeartMate 3 may be a risk factor for persistent lead dysfunction.

Hu et al.^16^ reported that patients with >50% decreased sensing had lower estimated glomerular filtration rate, were more likely to have undergone concomitant tricuspid valve surgery or RV assist device implantation, or had cardiac tamponade or an unplanned return to the operating room, albeit this study did not include a multivariable analysis. We could not identify these as predictive risk factors for a persistent decrease in sensing. In their analysis, 7 of 33 patients showed sensing dysfunction, although 2 were temporary. They did not divide dysfunction into persistent and transient, which differs from this study. We could not identify any risk factors for any sensing dysfunction, including persistent and temporary dysfunction.

Persistent lead dysfunction did not affect patient mortality. Moreover, almost all cases of lead dysfunction are managed conservatively. LVAD patients require anticoagulation and antiplatelet drugs; therefore, their risk of bleeding is high.^43^ Previous studies have reported a high incidence of bleeding complications in patients with LVADs undergoing implantation of new CIEDs or generator replacement.^44^^,^^45^ If possible, invasive treatment should be avoided in patients with LVAD, and our results support the idea that unnecessary intervention for lead dysfunction is not required, even with persistent lead dysfunction.

### STUDY Limitations

Our study had several limitations. First, this study was limited by its retrospective and nonrandomized nature. Some patients with LVAD did not have CIED interrogation dates. We could not include all patients because of insufficient data. Second, the timing of ICD interrogation differed from patient to patient, although almost all patients underwent this once every 6 months. However, differences in the timing of the ICD interrogation would affect the precise evaluation of the timing of lead dysfunction. The changes in parameters generally occurred gradually, and the difference in interrogation timing in our study would not affect the statistical results. Third, patients who underwent LVAD implantation for BTT in Japan were required to wait longer for heart transplantation than those in Western countries. Therefore, the backgrounds of patients who receive long-term LVAD support differ from those of patients in Western countries. Although the frequency of LVAD implantation as DT would be lower than that in previous studies performed in Western countries, cardiac functions and other background details were not very different. Fourth, this study offers modest discriminative ability, with an AUC of 0.67. Although this level of performance does not support immediate clinical implementation, this research was designed as an exploratory analysis rather than a definitive risk-prediction model. Fifth, although survival differences between patients with and without lead dysfunction were analyzed, this was performed after LVAD implantation and lead dysfunction had occurred later. To assess the impact of lead dysfunction on mortality, a longer follow-up duration after lead dysfunction is required, and further studies are needed.

## Conclusions

Over 46.2 months, ICD lead dysfunction was observed in up to 72.9% of patients, of which approximately 40% occurred more than a year after LVAD implantation, and two-thirds showed persistent dysfunction. Later occurrence of lead dysfunction may be related to persistent lead dysfunction.

## Funding support and author disclosures

Dr Miyamoto received funding/grants received from 10.13039/100019341Medtronic Japan Co, Ltd, Abbott Japan LLC, and Boston Scientific Japan Co, Ltd. Dr Kusano reports funding/grants received from 10.13039/100019341Medtronic Japan Co. Ltd and he received Speakers Bureaus from 10.13039/100019341Medtronic Japan Co, Ltd, outside of the submitted work. Dr Ishibashi received honoraria for lectures from 10.13039/100019341Medtronic Japan Co, Ltd, 10.13039/100015643JAPAN LIFELINE Co, Ltd, and Biotronik Japan Inc outside of the submitted work. Drs Oka and Ueda received honoraria for lectures from 10.13039/100019341Medtronic Japan Co, Ltd, outside of the submitted work. Dr Wakamiya received honoraria for lectures from 10.13039/100019341Medtronic Japan Co, Ltd and Biotronik Japan Inc outside of the submitted work. Dr Miyamoto received honoraria/Speakers Bureaus from 10.13039/100019341Medtronic Japan Co, Ltd, Abbott Japan LLC, and Boston Scientific Japan Co, Ltd outside the submitted work, and is affiliated with a department endowed by 10.13039/100019341Medtronic Japan Co, Ltd outside the submitted work. All other authors have reported that they have no relationships relevant to the contents of this paper to disclose.
