# Supplementary material for: Long-Term Implantable Cardioverter Defibrillator Lead Dysfunction After Left Ventricular Assist Device Implantation
Source: JACC Adv. 2025 Oct 24;4(11):102258. doi: 10.1016/j.jacadv.2025.102258 (PMC12595381; doi:10.1016/j.jacadv.2025.102258)
Supplement: Supplemental Table 1 [file mmc1.docx]

| SUPPLEMENTAL TABLE 1. Univariable and Multivariable models for predictive risk factors of persistent lead dysfunction (N=84) among all included leads (N=170) |
| --- |

|  | Univariable analysis | | Multivariable analysis | |
| --- | --- | --- | --- | --- |
| Predictors | Hazard Ratio (95% CI) | p value | Hazard ratio (95% CI) | p value |
| Age, per 1 year increase | 1.004 (0.987-1.013) | 0.65 |  |  |
| Female | 1.563 (0.980-2.868) | 0.060 | 1.641 (1.025-2.629) | 0.039 |
| Body mass index per 1 kg/m^2^ increase | 0.972 (0.823-1.012) | 0.25 |  |  |
| LVEF per 1% increase | 0.999 (0.971-1.027) | 0.99 |  |  |
| Hypertension | 0.893 (0.273-2.756) | 0.85 |  |  |
| Diabetes mellitus | 0.916 (0.506-1.658) | 0.77 |  |  |
| Chronic kidney disease | 1.160 (0.755-1.783) | 0.50 |  |  |
| eGFR per 1 mL/min/1.73m^2^ increase | 0.998 (0.989-1.007) | 0.74 |  |  |
| Ischemic cardiomyopathy | 2.171 (0.992-4.747) | 0.052 | 2.297 (1.040-5.071) | 0.039 |
| Prior sternotomy | 1.367 (0.811-2.305) | 0.24 |  |  |
| ICD lead manufacturer |  |  |  |  |
| Medtronic ICD lead | 1.094 (0.712-1.681) | 0.68 |  |  |
| Abbott ICD lead | 0.764 (0.448-1.302) | 0.32 |  |  |
| Boston ICD lead | 1.071 (0.603-1.902) | 0.82 |  |  |
| Biotronic ICD lead | 1.056 (0.507-2.197) | 0.88 |  |  |
| Intermacs score |  |  |  |  |
| 1 | 0.863 (0.446-1.671) | 0.66 |  |  |
| 2 | 0.818 (0.467-1.432) | 0.48 |  |  |
| 3 | 1.166 (0.752-1.801) | 0.49 |  |  |
| 4 | 1.123 (0.561-2.251) | 0.74 |  |  |
| Bridge to transplantation | 1.030 (0.473-2.243) | 0.94 |  |  |
| Time from lead implantation to LVAD per 1 month increase | 1.000 (0.994-1.005) | 0.80 |  |  |
| Type of LVAD |  |  |  |  |
| HeartMate 3 | 1.669 (1.034-2.693) | 0.035 | 1.635 (1.012-2.641) | 0.044 |
| HeartMate Ⅱ | 1.110 (0.722-1.708) | 0.63 |  |  |
| Jarvik2000 | 0.621 (0.227-1.700) | 0.35 |  |  |
| HVAD | 0.654(0.284-1.503) | 0.32 |  |  |
| EVAHEART | 0.621 (0.285-1.353) | 0.23 |  |  |
| DuraHeart | 0.447 (0.062-3.220) | 0.42 |  |  |
| Concomitant cardiac surgery | 0.997 (0.630-1.577) | 0.99 |  |  |

CI=confidential interval; LVEF = left ventricular ejection fraction; eGFR = estimated glomerular filtration rate; ICD = implantable cardioverter defibrillator; LVAD = left ventricular assist device.
